# Vitamin D and Diabetic Retinopathy

**DOI:** 10.3390/ijms241512014

**Published:** 2023-07-27

**Authors:** Antonela Gverović Antunica, Ljubo Znaor, Mira Ivanković, Velibor Puzović, Irena Marković, Snježana Kaštelan

**Affiliations:** 1 Department of Ophthalmology, General Hospital Dubrovnik, 20000 Dubrovnik, Croatia; 2 Clinical Department of Ophthalmology, University Hospital Center, 21000 Split, Croatia; 3 Department of Neurology, General Hospital Dubrovnik, 20000 Dubrovnik, Croatia; 4 Department of Pathology, General Hospital Dubrovnik, 20000 Dubrovnik, Croatia; 5 Department of Ophthalmology, University Hospital Dubrava, 10000 Zagreb, Croatia

**Keywords:** vitamin D, diabetic retinopathy, hypovitaminosis, calcitriol, supplementation

## Abstract

Diabetic retinopathy (DR) is the most common eye disease complication of diabetes, and hypovitaminosis D is mentioned as one of the risk factors. Vitamin D2 (ergocalciferol) and vitamin D3 (cholecalciferol) are the best-known forms of vitamin D. Calcitriol (1,25-dihydroxycholecalciferol) is the active form of vitamin D, with the sun being one of its main sources. Vitamin D is synthesized in the skin by exposure to sunlight without protective factors, but care must be taken to avoid the development of sunburn. It not only plays an important role in maintaining healthy bones and immune system but has also been highlighted in numerous studies to have an influence on various diseases, including diabetic retinopathy. A large number of people suffer from vitamin D hypovitaminosis worldwide, and diagnosis is made by measuring the concentration of 25-hydroxyvitamin D (25(OH)D) in serum. Its deficiency can cause numerous diseases and, as such, supplementation is necessary. Clinical studies have proven the effectiveness of vitamin D supplementation in the treatment of diabetic retinopathy, but with a doctor’s recommendation and supervision due to possible negative side effects.

## 1. Introduction

Vitamins and minerals play an important role in the growth, development, and maintenance of a healthy body [1,2,3]. Vitamins are usually ingested and supplemented through food as they cannot be produced by the body itself. However, some vitamins can be synthesized in smaller amounts from provitamins (e.g., the production of vitamin D from provitamin D with the help of ultraviolet rays, or sunlight [4].

Vitamins are divided into two main groups: fat-soluble (A, D, E, and K) and water-soluble (C and B-complex) [1]. Insufficient dietary intake, inadequate absorption, and complete or partial deficiency of vitamins (hypovitaminosis or avitaminosis) can lead to dysfunction in the body and damage to numerous organs, which in turn can result in various diseases. Excess amounts of fat-soluble vitamins accumulate in the body and can have harmful effects, while excessive amounts of water-soluble vitamins are excreted in the urine, which poses less risk of harmful effects. Vitamins should only be taken on the advice of a physician or pharmacist to avoid uncontrolled intake [1].

There are prescribed daily doses of vitamins and minerals depending on the sex, age, and condition of the consumer in terms of childhood, pregnancy, or lactation. The activity of vitamins can be expressed in international units (IU), milligrams (mg), and micrograms (µg) [4].

In the past, significant numbers of children suffered from rachitis. Children living in areas with moderate temperatures and in urban areas were most commonly affected. There were two theories for curing rachitis [4]: Some scientists claimed that the body must be exposed to sunlight and fresh air, while others claimed that the diet must contain a certain ingredient that would lead to the cure. Both theories were confirmed in 1919, when the first papers on rachitis were published, and the first research was conducted on an animal model that showed the possibility of treating rachitis in 1924. Subsequently, the structures of calciferol and cholecalciferol, which are further processed in the body into active components, were elucidated [4]. Diabetic retinopathy is becoming a major health problem with possible serious complications and limited normal functioning [5,6]. Various risk factors are known, as well as a correlation with vitamin D, and such findings are presented in this article. Several ways in which vitamin D may be linked to diabetic retinopathy are its anti-inflammatory effect, antioxidant effect, blood pressure regulation, and impact on insulin sensitivity.

## 2. Vitamin D

Vitamin D (VD) is an essential micronutrient often referred to as “the sunshine vitamin” [7]. Although it is classified as a vitamin, it is actually a hormone due to its steroid structure and mechanism of action [4]. It is synthesized in the skin when exposed to sunlight, and when it is not adequately produced, it must be ingested through diet [7]. VD regulates calcium homeostasis in the body and also regulates plasma Ca^2+^ concentration by cooperating with parathyroid hormone (PTH) [7]. The VD group includes seven vitamins that differ in the structure of the side chain at position 17. Vitamin D consists of a mixture of vitamin D2 (ergocalciferol) and D3 (cholecalciferol), which are the most effective forms for therapeutic purposes. The provitamin for vitamin D2 (ergosterol) is of plant origin, while the provitamin for vitamin D3 (7-dehydrocholesterol) is of animal origin [6]. VD promotes the absorption of calcium and phosphate and stimulates normal growth and mineralization of the bones and teeth [8]. In addition to its importance in bone health, numerous studies suggest that VD promotes musculoskeletal and immune health and contributes to the prevention and treatment of cardiovascular disease and certain cancers [8]. A deficiency of VD can also lead to obesity, hypertension, type 1 and type 2 diabetes, autoimmune diseases, and depression [9]. Other diseases associated with a deficiency of VD include multiple sclerosis, rheumatoid arthritis, and Alzheimer’s disease. Mental health has also been shown to be affected by a deficiency of VD [10,11].

VD belongs to the group of fat-soluble vitamins. The body absorbs it through foods that contain VD, through dietary supplements, or through sunlight, which stimulates synthesis in the liver and kidneys [7]. VD is inert in the body until it undergoes a two-step hydroxylation process in the liver and kidneys to become biologically active. It is most commonly associated with bone health and the prevention of osteomalacia and rachitis [12]. An important finding is that the VD gene is expressed in living cells and that approximately 3% of the human genome is under its control [13]. Recent data therefore points to the beneficial effects of VD on the immune and cardiovascular systems, as well as on the prevention of autoimmune and malignant diseases [14]. Increasing public awareness of the health benefits of VD may increase the risk of toxic effects if the vitamin is taken in higher doses than recommended for age and body weight. Fatty fish such as tuna, mackerel, and salmon, as well as fish liver oils, are among the richest sources of VD. Small amounts are found in cheese, beef liver, and egg yolks. Some mushrooms also provide VD in varying amounts. It is also commercially available in various forms, including water- and oil-soluble capsules, concentrates, tablets, chewing gum, sprays, injections, and oral drops, which are the only acceptable form of administration for infants. For children over 1 year of age and adults, the recommended daily dose varies depending on age, gender, and other factors [15]. The process of VD synthesis in the body begins with the conversion of 7-dehydrocholesterol, a precursor molecule found in the skin, into previtamin D3 by UVB radiation, which then undergoes a thermal isomerization process to form vitamin D3 [13]. Vitamin D3 is then transported to the liver where it is hydroxylated to 25-hydroxyvitamin D3 (25(OH)D3), the primary form of VD found in the blood. From the liver, 25(OH)D3 is then transported to the kidneys, where it is further hydroxylated to form 1,25-dihydroxyvitamin D3 (1,25(OH)2D3), also known as calcitriol, which is the active form of VD [16]. VD supplements are a convenient way to increase the intake of VD when it is insufficient from sunlight or food [13]. The recommended daily dose of VD varies by age, with the Institute of Medicine (IOM) recommending the following [15]:

Infants 0–12 months: 400–1000 IU/day;

Children 1–13 years: 600–1000 IU/day;

Adolescents 14–18 years: 600–1000 IU/day;

Adults 19–70 years: 600–1000 IU/day;

Adults over 70 years: 800–1000 IU/day.

High amounts of VD can cause negative side effects such as nausea, loss of appetite, vomiting, constipation, and weight loss [17]. In addition, people with certain medical conditions such as sarcoidosis, tuberculosis, lymphoma, and hyperparathyroidism should avoid taking VD without consulting a physician [15]. VD supplements do not provide the same benefits as natural sunlight and a balanced diet and therefore should not be used as a substitute for these sources. The amount of UVB radiation can vary depending on the time of day, season, location, and use of sunscreen [18,19]. In addition, certain factors such as age, skin pigmentation, and the use of certain medications can affect the body’s ability to synthesize VD. Therefore, it is important to monitor VD levels and consider supplementation if levels are low [20].

## 3. Vitamin D and Diabetic Retinopathy

Diabetes mellitus is a chronic metabolic disorder of carbohydrates, fats, and protein caused by a relative or absolute lack of insulin [21]. According to etiology and characteristics, it is divided into diabetes type 1 and diabetes type 2. Regardless of cause, it comes as a result of hyperglycemia, i.e., an increase in the concentration of glucose in the blood. Long-term elevated blood glucose levels lead to changes in the endothelium of large blood vessels (macroangiopathic changes) that are manifested by ischemic heart disease and cerebral and peripheral vascular disease. There are also changes in the endothelium of smaller blood vessels (microangiopathic changes) characterized by kidney damage—diabetic nephropathy and damage to the peripheral nerves—diabetic neuropathy, and eye vascular changes, with the largest clinical significance being of the retina, i.e., diabetic retinopathy [22]. Diabetes is a significant public health problem due to increasing morbidity and mortality related to its complications. According to the World Health Organization, about 422 million people suffer from diabetes around the world, mostly in developed and developing countries. Although diabetes occurs at any age, microangiopathic complications are often manifested several tens of years after diagnosis, depending on the type of disease and its therapeutic control [23,24,25].

Diabetic retinopathy (DR) is the most common eye disease complication of diabetes and also the most common microvascular complication of diabetes in general. The primary mechanism responsible for vision loss in diabetic retinopathy is diabetic macular edema (DME), defined as vascular leakage and consequent fluid accumulation that damages the center of the macula. The second mechanism is characterized by the appearance of neovascularization in the optic disc or retina resulting from ischemia, which is called proliferative diabetic retinopathy (PDR) [26,27,28].

Working in Toronto in 1921, Bantig and Best discovered a hormone created in the beta cells of the islets of Langerhans—insulin. On 11 January 1922, the first patient with diabetes received insulin, prompting a dramatic revolution in the treatment and prolongation of the lifespan of diabetic patients and more pronounced manifestations of late diabetic complications, with angiopathy as a common denominator. At the end of 1963, the insulin molecule was successfully synthesized [22].

Experimental evidence suggests that hyperglycemia is a “trigger” cascade biochemical process that leads to vascular dysfunction and early structural changes in the vascular wall [22].

Endothelial vascular dysfunction is a fundamental etiological factor in the development of various clinical complications, including DR and diabetic nephropathy. Endothelial hypoxia is the cause of hyperpermeability.

Known risk factors for DR include duration of diabetes, blood glucose level, body mass index (BMI), and hypertension [29,30,31]. However, recent studies have also cited serum levels of VD as a potential risk factor [32,33,34].

It is known that lower serum concentrations of VD are associated with an increased risk for diseases such as myopia, age-related macular degeneration, and dry-eye syndrome [32].

In Table 1, serum levels and corresponding status of 25(OH)D concentrations are presented.

Zoppini et al. demonstrated a significant association between serum levels of 25(OH)D and the prevalence of DR in a study of 715 patients with type 2 diabetes [35]. Alcubierre et al. reached the same conclusion that patients with advanced DR had lower serum 25(OH)D concentrations compared to patients without DR [36]. Zhao et al. demonstrated in a meta-analysis that patients with type 2 diabetes and VD deficiency (i.e., serum 25(OH)D below 20 ng/mL) have a significantly increased risk of DR. Their results indicate an association between VD deficiency and increased risk of DR in type 2 diabetic patients [37]. In a 2000 study by Aksoy et al., an inverse relationship was found between the presence and severity of DR and VD concentrations. The lowest concentrations were observed in proliferative DR, while the highest concentrations were found in patients with diabetes and without DR [38]. A 2011 study of over 500 patients found that deficiency of VD doubled the prevalence of DR in patients with type 1 diabetes [39]. Similarly, a retrospective study of nearly 1000 patients from the NHANES study showed that the prevalence of severe and mild DR was higher in patients with poor disease control and hypovitaminosis D than in patients with adequate VD levels [40].

A large number of studies suggest an inverse correlation between the concentration of VD and DR but do not completely explain the cause–effect relationship. It is not entirely clear whether low VD levels are the cause of DR or whether DR decreases VD concentration due to decreased physical activity and sun exposure [41,42,43,44,45,46]. Recent experimental studies suggest that VD protects against DR through its anti-inflammatory and anti-angiogenic properties. There is evidence that VD plays a role in the pathogenesis of DR by affecting the immune system and inflammatory cytokines such as tumor necrosis factor (TNF) α and TNF β [47]. Proinflammatory leukins such as interleukin 6 (IL-6) are increased in patients with type 2 diabetes, and VD has been shown to reduce the production of several proinflammatory cytokines [48,49,50]. VD has an anti-inflammatory effect by reducing the proliferation of T helper cells and cytotoxic T cells as well as the formation and activity of natural killer cells. Inhibition of angiogenesis is one of the pathways in which VD is associated with DR. A recent study found that deficiency of VD is associated with vascular endothelial dysfunction by reducing vascular endothelial growth factor (VEGF) expression, inhibiting endothelial cell proliferation, and reducing platelet-derived growth factor (PDGF) expression. There are studies showing that VD inhibits retinal neovascularization in vivo and in vitro. In addition, VD decreases the transcriptional activity of hypoxia-inducible factor-1 (HIF-1) [51,52]. It has been shown that VD also inhibits the gene expression of matrix metalloproteinases (MMPs), which have an impact on the development of DR [53]. Low circulating levels of VD in diabetic patients may lead to decreased neuroprotective effects and optic neuropathy. Gungor et al. compared retinal nerve fiber layer (RNFL) thickness in patients with early-stage DR with or without VD deficiency (<20 mg/mL) and demonstrated that mean RNFL was significantly reduced in patients with VD deficiency [54]. However, there are a small number of reports that do not confirm the association between the concentration of VD and the development of DR, such as a Chinese study conducted by Xiao et al. [55]. Similarly, an Indian study conducted by Reddy et al. shows an association between VD and diabetes but not with diabetic retinopathy [56].

Data on the effects of VD on macrovascular complications, cardiovascular risk, and diabetes complications are controversial. In a randomized controlled trial of over 25,000 patients, Manson et al. found that supplementation of VD for five years had no cardiovascular protective effect [57]. On the other hand, meta-analyses by Chowdhury and Mirhosseini suggest that hypovitaminosis D may reduce cardiovascular risk [58,59]. There are clinical studies that have shown that VD deficiency is associated with vascular damage, such as endothelial dysfunction assessed by brachial artery [60], flow-mediated dilation, and carotid intima-media thickness measurements [53]. Mechanistic studies have demonstrated the protective role of VD in vascular regeneration and highlight its crucial role in vascular protection [61]. In addition, VD has been reported to have a protective effect against cerebrovascular complications [51]. Long’s retrospective study of 1000 patients showed that the prevalence of severe and mild DR was higher in poorly controlled patients with hypovitaminosis D than in patients with normal VD levels [62]. A meta-analysis with more than 17,000 participants from 15 observational studies found that individuals with type 2 diabetes and VD deficiency are at increased risk of DR [63]. Another meta-analysis with over 10,000 participants from 14 observational studies showed a significant association between DR and VD deficiency where patients with DR showed a decrease in the levels of VD [64]. Table 2 shows the results of studies on the relationship between VD and DR.

An important influence in the pathogenesis of DR is attributed to the abnormal production of reactive oxygen species (ROS) and chronic inflammation [65]. Lu et al. have shown that the ROS/TXNIP/NLRP3 inflammasome pathway is the main mechanism in the development of DR and that NLRP3 inflammasome has an effect in vascular damage in advanced stages of the disease. NLRP3 is a cytosolic pattern recognition receptor (PRR) protein of 118 kDa and can be expressed in various cells, such as lymphocytes, neutrophils, macrophages, epithelial cells, osteoblasts, neurons, and dendritic cells [64]. The NLRP3 protein contains three domains: the C-terminal domain that contains leucine; the central NACHT domain that contains ATPase and mediates oligomerization; and the N-terminal pyrin domain that has a role in recruiting proteins to form the inflammasome complex. Similar to other inflammasomes, the NLRP3 inflammasome complex consists of a sensor (NLRP3 protein), an adapter (apoptosis-associated p, ASC), and an effector (caspase-1). Activated NLRP3 inflammasome mediates caspase-1 activation, cleaving proIL-1β and proIL-18 into their active forms [64].

Loukovaara et al. compared the concentrations of caspase-1 and IL-18 in the vitreous of eyes with PDR and NPDR and found that the levels were significantly higher in the vitreous of PDR eyes than in those with non-proliferative diabetic retinopathy. They concluded that NLRP3 plays an important role in the pathogenesis of PDR, which occurs in two forms, namely initial and activation [66]. Lu et al. presented a strong correlation between reduced vitamin D concentration in the vitreous and an increased NLRP3 inflammasome pathway in patients with PDR, which may be associated with the pathogenesis of PDR and have practical importance in its treatment [64].

The treatment of DR aims to slow down its progression, prevent vision loss, and improve visual function. The specific procedure of treatment may vary depending on the severity and stage of DR. A general outline of the treatment procedure follows:

1. Medical management: Proper blood sugar control, blood pressure management, and cholesterol control are essential to slow down the progression of the disease;
2. Laser photocoagulation;
3. AntiVEGF injections;
4. Corticosteroid implants;
5. Vitrectomy;
6. Other procedures (vitamin supplementation).

The choice of procedure is determined by the ophthalmologist according to the severity of the damage. It is important to note that the latest clinical trials have shown that the correction of VD in patients with diabetic macular edema can play an important role in improving macular edema but the effect is only visible after a few months. In diabetics, in addition to the regulation of blood glucose, hypertension, and lipid levels, the level of VD should therefore definitely be analyzed and, in cases of reduced concentration, should be supplemented [67].

## 4. Conclusions

DR is a serious condition that occurs in people with diabetes and is associated with damage to the blood vessels in the retina of the eye. VD is an important nutrient that performs many functions in the body, including regulating the immune system as well as inflammatory and angiogenic processes. According to most studies, there may be a link between VD deficiency and an increased risk of developing diabetic retinopathy. Although the association between VD and diabetic retinopathy is clear, the underlying pathophysiologic mechanisms are not completely certain or proven. Recent research has shown vitamin D is not only important in bone metabolism but also acts as a strong antioxidant that significantly reduces the formation of free radicals, has an anti-inflammatory effect, and modulates autophagy and apoptosis, meaning vitamin supplementation should be useful in reducing the damaging effects of free radicals in DR [67,68]. Further research, including randomized controlled trials, are needed to better understand this relationship and to determine whether VD supplementation has a genuine therapeutic effect in individuals with DR. In any case, it is important for people with diabetes to monitor their eye health regularly and to take all recommended preventive measures and treatments as directed by their physician. It is also important to maintain a balanced diet, to consult a qualified health professional if there is any uncertainty or if supplementation of VD or other nutrients is required, to undertake physical activity, and to ensure moderate sun exposure, especially nowadays when we spend most of our time indoors in front of the TV or computer.

## Figures and Tables

**Table 1:** Serum 25-hydroxy vitamin D concentrations and status.

Concentration	Status
<10 ng/mL	Severely deficient
10–20 ng/mL	Deficient
20–30 ng/mL	Insufficient
≥30 ng/mL	Normal
≥100 ng/mL	Possible toxicity

**Table 2:** Studies on the relationship between VD and DR.

First Author	Year	Country	Study Design	Sample Size	Main Finding
Harleen Kaur [39]	2011	Australia	Cross-sectional	517 patients with type 1 diabetes mellitus	VD deficiency is associated with an increased prevalence of retinopathy in young people with T1DM. DR is associated with diabetes duration and HbA1c.
Snježana Kaštelan [29]	2013	Croatia	Cross-sectional	545 patients with type 2 diabetes	Progression of DR increased significantly with higher BMI.
Martina Tomić [30]	2013	Croatia	Cross-sectional	107 patients with type 2 diabetes	Diabetes duration and prolonged poor glycemic control are the main predictors of DR in patients with type 2 diabetes.
Snježana Kaštelan [31]	2014	Croatia	Cross-sectional	176 patients with type 1 diabetes: group 1 (no retinopathy; *n* = 86), group 2 (mild/moderate nonproliferative DR; *n* = 33), and group 3 (severe/very severe NPDR or proliferative DR; *n* = 57).	DR progression is correlated with diabetes duration, HbA1c, hypertension, total cholesterol, and the presence of nephropathy.
Giacomo Zoppini [35]	2015	Italy	Cross-sectional	715 outpatients with type 2 diabetes	Inverse and independent relationship between circulating 25(OH)D3 levels and the prevalence of microvascular complications in patients with T2DM.
Nuria Alcubierre [36]	2015	Spain	Observational case-control	Two groups of patients were selected: 139 and 144 patients with and without retinopathy	Association of vitamin D deficiency with the presence and severity of diabetic retinopathy in type 2 diabetes.
G Bhanuprakash Reddy [56]	2015	India	Cross-sectional case-control	82 T2DM with DR patients and 99 healthy controls	Association between vitamin D deficiency and type 2 diabetes, but not specifically with retinopathy.
Adem Gungor [54]	2015	Turkey	Prospective	50 VDD with DR patients and 50 VDD without DR patients	VD acts as a neuroprotective component for optic nerves. Low serum 25(OH)D concentrations contribute to RNFL thinning in patients with early-stage VDD DR.
Markus Herrmann [41]	2015	Australia, New Zealand, and Finland	Multinational, double-blind, placebo-controlled trial	9795 patients with type 2 diabetes	Increased risk of macrovascular and microvascular disease events in T2DM are associated with low blood 25(OH)D_3_.
Beteal Ashinne [42]	2018	India	Retrospective	3054 patients with type 2 diabetes mellitus	Lower serum 25(OH)D3 is associated with a higher severity of DR, and the presence of vitamin D deficiency is associated with a twofold increased risk of PDR.
Hülya Aksoy [38]	2000	Turkey	Cross-sectional	66 patients with type 2 diabetes mellitus	Inverse relationship between severity of retinopathy, neovascularization, and serum 1,25(OH)2D3 concentrations, which were lowest in PDR patients and highest in diabetic patients without retinopathy.
Abdulbari Bener [43]	2018	Turkey	Cross-sectional	638 patients with type 2diabetes mellitus	Vitamin D deficiency is considered a risk factor for DR and hearing loss in diabetics.
Gauhar Nadri [44]	2019	India	Cross-sectional	72 patients with DM, 24 without DR, 24 with NPDR, and 24 with PDR	Serum vitamin D levels ≤ 18.6 ng/mL serve as a sensitive and specific indicator of proliferative disease in patients of DR. VD is a significant predictor of diabetic retinopathy severity.
Jing Yuan [45]	2019	China	Cross-sectional	889 patients with type 2 diabetes	Vitamin D deficiency is significantly associated with risk of PDR.
Abdulhalim Senyigit [46]	2019	Turkey	Cross-sectional	163 patients with type 2 diabetes and 40 controls	Low serum 25-OHD levels are associated with the development of diabetes and complications. Serum 25(OH)D levels were significantly lower in all patients than in the control group. The 25(OH)D levels of patients with complications were lower than those of patients without complications.
Ying Xiao [55]	2020	China	cross-sectional study	4284 patients with type 2 diabetes mellitus	DR is associated with VDD status and the association remains after adjustment for age, sex, and other demographic and physical measures.
Wei-Jing Zhao [37]	2021	China	cross-sectional study,	815 patients with type 2 diabetes mellitus	Patients with type 2 diabetes and VD deficiency (<20 ng/mL) have a significantly increased risk of DR.

## Data Availability

Not applicable.
